# Assessment of transient elastography (FibroScan) for diagnosis of fibrosis in non-alcoholic fatty liver disease: A systematic review and meta-analysis

**Published:** 2016

**Authors:** Seyed-Abbas Hashemi, Seyed-Moayed Alavian, Mohammad Gholami-Fesharaki

**Affiliations:** 1 Baqiyatallah Research Center for Gastroenterology and Liver Diseases, Baqiyatallah University of Medical Sciences, Tehran, Iran; 2 Middle East Liver Diseases Center (MELD), Tehran, IR Iran.; 3 Department of Biostatistics, Faculty of Medical Sciences, Tarbiat Modares University, Tehran, Iran.

**Keywords:** Transient elastography, Fibro scan, Fibrosis, Non-alcoholic fatty liver disease

## Abstract

**Background:**

Transient elastography (TE) is a new modality for the diagnosis of liver fibrosis caused by various etiologies. This study was conducted to determine the accuracy of TE in detecting the different stages of liver fibrosis in non-alcoholic fatty liver disease (NAFLD) patients.

**Methods:**

MEDLINE/PubMed, Embase, Ovid, Cochrane Library, American College of Physicians (ACP) Journal Club, Google Scholar, Database of Abstracts of Reviews of Effects, and Web of Science that evaluated the liver stiffness by means of TE and liver biopsy were enrolled in this systematic review and meta-analysis. Published articles were extracted from 2002 to March 2015.

**Results:**

A total of 7 articles from 114 papers were included which consisted of 698 patients. The results indicated that when F ≥3, the outcomes were 93.7% (95% confidence interval (CI): 92-95.5), 91.1% (95% CI: 89-93.2), 82.4% (95% CI: 79.9-84.9), and 95.9% (95% CI: 94.4-97.4) for sensitivity, specificity, positive predictive value (PPV) and negative predictive value (NPV), respectively. With fibrosis stage ≥4, it has reached the sensitivity of 96.2 % (95% CI: 94.5-97.8), a specificity of 92.2% (95% CI: 89.9-94.6), a PPV of 5.5% (95% CI: 51.2-59.8) and NPV of 98.5% (95% CI: 97.4-99.5).

**Conclusion:**

We concluded that as the pathological fibrosis increases, the sensitivity, specificity and NPV of TE in the diagnosis of fibrosis improves in NAFLD patients. TE can be considered as a unique alternative instead of liver biopsy in NAFLD patients and it has an important role in the exclusion of liver cirrhosis. More studies are required to confirm the results.

Non-alcoholic fatty liver disease has been considered as one of the main etiology of chronic liver disease globally (1, 2). Non-alcoholic fatty liver disease (NAFLD) has two main subgroups including non-alcoholic fatty liver (NAFL) and non-alcoholic steatohepatitis (NASH). NASH has been defined as steatosis with inflammation, hepatocellular injury and possible fibrosis. Sometimes the final outcomes of NASH are cirrhosis and hepatocellular carcinoma. NAFL and NASH are different spectrum of the same histological disease (3, 4). The gold standard tool to diagnose NAFLD is liver biopsy and histological study and various stages are clarified according to histopathological investigations (3). There are some limitations for liver biopsy like invasive nature, complications, low level of individuals' satisfaction and sampling variation (5). 

Pain and hypotension are major complications of liver biopsy and can lead to increased length of hospital stay and cost (6). The mortality rate after percutaneous liver biopsy has been reported as 1 in 10000 to 1 in 12000 (7). Therefore performing continuous liver biopsy for follow-up is practically impossible (8).

There were many investigations which tried to find methods to identify NASH including imaging evaluations and blood tests but still these procedures could not diagnose it well (9). Consequently, investigators are trying to find non-invasive valuable procedure in the diagnosis of liver stiffness/fibrosis. In this regard, application of Fibroscan (transient elastometer) (Echo Sens, Paris, France) is a device that can examine liver stiffness (10, 11). Diagnosis of liver stiffness and fibrosis by this method was reported previously (12, 13). Thus serial evaluation of liver stiffness can provide evidence about the progression of liver diseases like NASH (14). Transient elastography (TE or FibroScan ® - FS) was first described in France (15, 16), then in other parts of the world (17-22). 

Lately published studies have shown that TE is valuable in detecting fibrosis and cirrhosis in chronic hepatitis patients (23-25) but with some limitations in overweight patients (26, 27). Nonetheless, the role of TE has not been well established in NAFLD/NASH patients due to non-optimal function of TE in overweight and obese people which is prevalent in people with NAFLD. Therefore, the aim of the current investigation was to assess the accuracy of TE in detecting various stages of fibrosis in NAFLD patients.

## Methods

**Literature Search** **and quality assessment: **Systematic review of the literature published in English about transient elastography for the diagnosis and staging of NAFLD⁄NASH was performed with the help of the following: MEDLINE/PubMed, Embase, Ovid, Cochrane Library, and American College of Physicians (ACP) Journal Club, Google Scholar, Database of Abstracts of Reviews of Effects, and Web of Science. We evaluated the sources between 2002 (when TE was first introduced) to March 2015. The search terms used were (FibroScan, transient elastography, elastography and liver, liver stiffness, noninvasive method and liver stiffness, liver fibrosis, NASH, NAFLD, fatty liver, steatosis, assessment, staging). Only full length papers were enrolled for primary assessment and case reports, review articles, meta-analysis and systematic review papers and letter to editors were removed. For quality, we used the quality assessment of diagnostic accuracy studies (QUADAS) checklist (28). Each paper meeting the inclusion criteria was analyzed by 2 independent reviewers (Seyed Moayed Alavian and Seyyed Abbas Hashemi). Two independent reviewers (Seyed Moayed Alavian and Seyyed Abbas Hashemi) studied all candidate papers, and they retrieved the full texts of published articles that could not be evaluated with the title and abstract alone. Articles which reported information needed for the meta-analysis were included in this study. We included 7 full papers in which liver biopsy was listed as the reference for the assessment of TE for fibrosis in NAFLD patients.

**Figure 1:**
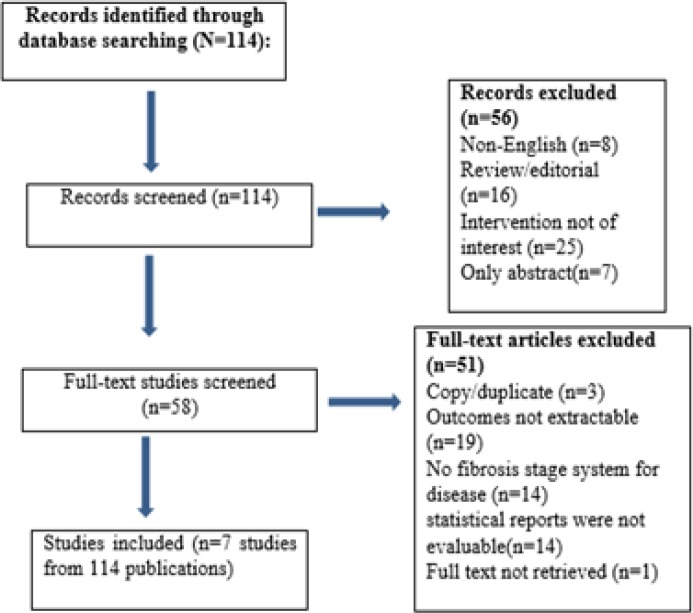
Flow sheet of the included databases

**Study Inclusion criteria: **Articles were selected according to the preferred reporting items for systematic reviews and meta-analyses (PRISMA) statement (29). The inclusion criteria for the articles included:1) investigations that only examined the NAFLD⁄NASH patients, 2) evaluated the performance of TE to establish liver fibrosis stages, 3) used reliable liver staging system, 4) examined the diagnostic value of TE and expressed sensitivity, specificity, positive predictive values (PPVs), or negative predictive values (NPVs) for the diagnosis of fibrosis stage based on certain cutoff TE values, 5) studies with at least 30 subjects to provide enough evidence to approve the outcomes (figure 1).

**Study exclusion criteria: **Our exclusion criteria were the following:1) if the article did not include the patients with NAFLD⁄NASH, 2) did not use a fibrosis staging system,3) did not report sensitivity, specificity, PPVs, or NPVs,4) articles that were not in English, 5) only the abstract form existed, 6) review or other types of paper which were not in the opinion of investigators (research studies which investigated NAFLD in hepatitis B virus (HBV), hepatitis C virus (HCV) patients and other etiologies.

**Data extraction: **The authors extracted the required information from articles independently. The following data were age, sex, and body mass index (BMI), number of cases, the area under the receiver operating characteristic curve (AUROC); the median liver stiffness; liver biopsy size and stage: sensitivity, specificity, PPV and NPV. We used “blinding” “separation task” method to decrease bias.

**Statistical analysis and meta- analysis: **The mean value and standard deviation were calculated for numerical variables with normal distribution. Qualitative variables were indicated as numbers and percentages. 95% confidence intervals were calculated for each predictive test and a p-value < 0.01 was considered as significant for each statistic testing. All collected data were entered into stata metan software Version 11 (Stata Corporation, College Station, TX, USA). The I^2^ statistic was used to evaluate the extent of variability attributable to statistical heterogeneity among articles. Heterogeneity was considered statistically significant when p heterogeneity was <0.1 or I^2^ more than 50%. If heterogeneity existed, data were analyzed using a fixed-effects model (Cochran’s Q test>0.1) and in the absence of heterogeneity, data were analyzed with random effects model (Cochran’s Q test<0.1). Heterogeneity between studies was evaluated with the Cochran Q-test and was considered to be present if the Q-test provided a p value of less than 0.10.

**Publication bias: **We used STATA to assess funnel plot asymmetry (to investigate publication bias) (30, 31) with both Begg's (32) and Egger's (33) methods and for meta-regression analysis.

## Results

A total of 114 full papers were evaluated. All articles were examined by S M A and S A H and approved for inclusion (selected articles overall scored highly on the QUADAS assessment). Finally, seven eligible articles with full text were found to be suitable and enrolled into the study. A total of 698 patients were included in this study (figure 1). Sensitivity analysis was done to evaluate variations and there were no variations in pooling effect. Demographic features of the current studies were indicated in table 1. Nobili et al. (34) showed that liver stiffness (LS) detected by TE more than 9 kPa was associated with high stage of fibrosis in pathology (table 2). Yoneda et al. (35) evaluated LS in 97 NAFLD patients. There were significant correlation between METAVIR (36) score and different stages (p<0.0001) (table 2). Based on the METAVIR scoring system, fibrosis is staged on a scale from F0 to F4, including: F0: no fibrosis; F1: portal fibrosis, without septa; F2: few septa; F3: many septa without cirrhosis; and F4: cirrhosis, respectively.

**Table1:** Main patients’ characteristics of the research populations in the studies that were enrolled in this paper; BMI (body mass index): Numerical variables with normal distribution were indicated as mean value ± standard deviation, while variables with non-normal distribution were reported as median values and range intervals

**Study**	**Year**	**Patient**	**Male**	**Female**	**Age**	**BMI**
Lupşor et al. (37)	2010	72	51 (70.8%)	21 (29.2%)	42 (20-69)	28.71 (20.96-41.53)
Wong et al. (French cohort) (8)	2010	128	74 (57.8%)	54(42.1%)	53±13	29.1 ±5.1
Wong et al. (Chinese cohort) (8)	2010	118	61 (51.7%)	57 (48.3%)	49 ±9	26.9± 3.4
Bokl et al. (38)	2012	30	-	-	-	
Yoneda et al. (35)	2008	97	40	57	51.8±13.7	26.6±4.2
Nobili rt al. (34)	2008	50	-	-	13.6±2.4	-
Gaia et al. (39)	2011	72	52	20	48 (24 - 65)	27.5 (21.1 -40.4)
Mahadeva et al. (40)	2013	131	69 (52.7)	62 (47.3)	49.9 ± 12.3	

**Table 2:** Concordance between fibrosis stages according to liver biopsy and TE in different investigations; Transient elastography (TE), fibrosis (F), Area under the Receiver Operating Characteristic Curve (AUROC), LB: Liver Biopsy, LS: Liver Stiffness, non-alcoholic steatohepatitis (NASH).

**Study**	**Diagnosis Of NASH**	**Scoring system**	**Liver Stiffness (kPa)**	**Liver Biopsy: Length (mm)/** **Portal Tracts (n)**	**Stage (n)**	**AUROC Value**	**Liver Stiffness (kPa) Median**	**Liver Stiffness (kPa) Cutoff** **Value**
Lupşor et al. (37)	liver biopsy	Brunt	2.80 to 16.90	11 (6-20) mm/ 11 (7-22) portal spaces	F0: 25 (34.7%)	F≥ 1: 0.879	F0: 4.90 (2.80-7.30)	F≥1:5.3
					F1: 29 (40.3%)	F≥ 2: 0.789	F1: 6.15 (4.80- 12.50)	F≥2:6.8
					F2: 13 (18.1%)	F≥ 3: 0.978	F2: 6.90 (3.30-16.90)	F3:10.4
					F3: 5 (6.9%)		F3: 14.00 (10.70-14.10)	
Wong et al. (French cohort)(8)	liver biopsy	Kleiner	9.7 ±9.7	24 ±8 mm/-	F0:14	F≥2: 0.87	F0:5.7 ±1.8	F≥2: 7.0
					F1:42	F≥3: 0.94	F1:6.8 ± 2.4	F≥3: 8.7
					F2:36	F=4: 0.94	F2:7.8 ±2.4	F=4: 10.3
					F3:21		F3:11.8 ± 5.2	
					F4;15		F4:25.1 ±17.1	
Wong et al. (Chinese cohort)(8)	liver biopsy	Kleiner	8.6 ±6.4	18±3 mm/-	F0:56	F≥2: 0.84	F0:5.7 ±1.8	F≥2: 7.0
					F1:33	F≥3: 0.92	F1:6.8 ± 2.4	F≥3: 8.7
					F2:9	F=4: 0.97	F2:7.8 ±2.4	F=4: 10.3
					F3:10		F3:11.8 ± 5.2	
					F4:10		F4:25.1 ±17.1	
Bokl et al (38)	liver biopsy	Kleiner	2.40 to 14.20	10 mm/-	F0: 18	Stage≥1:0.78	F0:4.76 ±1.58	Stage≥1:5.35
					F1:6	Stage≥2:0.78	F1:6.65±2.53	Stage≥2:5.35
					F2:4	Stage≥3:1.00	F2:6.85±3.62	Stage≥3:12.85
					F3:2		F3:14.05±0.21	
Yoneda et al. (35)	liver biopsy	Brunt	-	20 mm/-	F0: 18 (18.6%)	F≥2: 0.86	F0:4.850 ± 0.893	F≥1: 5.9
					F1: 28 (28.9%)	F≥3: 0.90	F1:7.382 ± 2.432	F≥2: 6.6
					F2: 24 (24.7%)	F≥ 4: 0.99	F2:9.283 ± 3.492	F≥3: 9.8
					F3: 18 (18.6%)		F3:13.333 ± 4.712	F≥ 4: 17.5
					F4; 9 (9.3%)		F4:25.344 ± 6.058	
Nobili et al. (34)	liver biopsy	Brunt	-			F≥2: 0.99	-	F≥2: 7.4
						F≥3: 1.0		F≥3: 10.2
						F≥ 4: -		F≥ 4: -
Gaia et al. (39)	liver biopsy	Brunt	6.6 (3.0–44.3)	25.2 mm (20–30.2)/-	F0: 23 (31.9)	F ≥1; 0.732	F0: 5.3 (3.0–9.7),	F0 vs F1234:5.5
					F1: 16 (22.2)	F≥2;0.758	F1: 6.15 (3.2–12.1)	F01 vs F234:7
					F2: 16 (22.2)	F≥3; 0.834	F2: 7.75 (4.3–13.9)	F012 vs F34:8
					F3: 8 (11.1)	F4; 0.862	F3: 6.5 (4.3–10.3)	F0123 vs F4:10.5
					F4; 9 (12.5)		F4: 11.9 (7.9–44.3)	
MAHADEVA et al. (40)	liver biopsy	Kleiner	9.0 ± 9.1	13.0 (8.0–15.0)/-	F0: 5 (3.8)	≥F2: 0.67	F0: 5.7 ± 1.4	≥F2: 6.65
					F1: 51 (39.0)	≥F3: 0.77	F1: 6.9 ± 2.3	≥F3: 6.95
					F2: 46 (35.1)	F4: 0.95	F2: 7.8 ± 3.5	F4: 10.60
					F3: 21 (16.0)		F3: 9.4 ± 7.4	
					F4; 8 (6.1)		F4; 29.1 ± 27.2	

In this regard, a study (37) indicated the median LSM values according to the fibrosis stages were: 4.90 kPa for F0; 6.15 kPa for F1; 6.90 kPa for F2 and 14.00 kPa for F3, with significant difference between stages (table 2). Bokl et al. (38) revealed that as the stage of fibrosis increases, the accuracy of TE improves. 

A study by Silvia Gaia et al. (39) examined 72 NAFLD patients using Brunt scoring system. They revealed liver stiffness (kPa). Cutoff value F0 vs F1234 was 5.5, F01 vs F234 became 7, F012 vs F34 was 8,F0123 vs F4 was 10.5 (table 2). An investigation (40) revealed that the accuracy of TE in describing the stage of fibrosis for ≥F3 was 0.77 (sensitivity 70.4% and specificity 66.6%) and for F4 was 0.95(sensitivity 87.5% and specificity 89.3%). Wong et al. (8) reported the role of TE in two ethnic groups including the Chinese and French population. They used Kleiner (41) ranking system. The results of French and Chinese population showed liver stiffness (kPa) cutoff values were F≥2: 7.0, F≥3: 8.7, F=4: 10.3 and F≥2: 7.0, F≥3: 8.7, F=4: 10.3, respectively (table 2). PPV, NPV, sensitivity, specificity with optimal cutoff and AUROC of all studies were summarized in table 3.

**Table 3:** Results of TE performance in quantifying fibrosis stages in NASH patients based on different studies (Optimal cutoff values for liver stiffness based on fibrosis stage); Positive Predictive Value (PPV) , Negative Predictive Value (NPV), Transient elastography (TE), fibrosis (F), area under the Receiver Operating Characteristic Curve (AUROC

	**Gaia (n:72)**	**Yoneda ** **(N 97)**	**Bokl ** **(N 30)**	**Nobili ** **(N 50)**	**Lupsor ** **(N 72)**	**Wong ** **(N 246)**	**Mahadeva** **(N 131)**
F≥ 1							
AUROC	0.776	0.92	0.78	-	-	-	-
Optimal cut-off (kPa)	5.5	5.9	5.35	-	-	-	-
Sensitivity (%)	84	86	83	-	-	-	-
Specificity (%)	57	88	72	-	-	-	-
PPV (%)	80	97	67	-	-	-	-
NPV (%)	62	59	87	-	-	-	-
F≥ 2							
AUROC	0.8	0.86	0.78	0.99	0.78	0.84	-
Optimal cut-off (kPa)	7	6.6	5.35	7.4	6.8	7.0	-
Sensitivity (%)	76	88	83	100	66.6	79	-
Specificity (%)	80	74	58	92	84.3	76	-
PPV (%)	75	79	33	80	60	70	-
NPV (%)	78	85	93	100	87	84	-
F≥ 3							
AUROC	0.7	0.90	1.00	1.0	0.978	0.93	0.77
Optimal cut-off (kPa)	8	9.8	12.85	10.2	10.4	8.7	7.10
Sensitivity (%)	65	85	100	100	100	84	70.4
Specificity (%)	80	81	100	100	97	83	66.6
PPV (%)	48	64	100	100	71	59	38.0
NPV (%)	86	93	100	100	100	95	88.7
F≥ 4							
AUROC	0.94	0.99	-	-	-	0.95	0.95
Optimal cut-off (kPa)	10.5	17.5	-	-	-	10.3	11.30
Sensitivity (%)	78	100	-	-	-	92	87.5
Specificity (%)	96	97	-	-	-	88	89.3
PPV (%)	70	75	-	-	-	46	34.3
NPV (%)	97	100	-	-	-	99	99.1

In the current investigation, the analysis of primary outcomes using fixed or random-effects tests revealed that for patients with fibrosis stage of ≥1, sensitivity, specificity, PPV, NPV were equal to 83.7%, 78.2%, 92.2%, and 65.6%, respectively. In cases with fibrosis stage of ≥ 2, sensitivity was 87.5%, specificity was 78.4%, PPV was 69.9% and NPV was 89.5%. When liver fibrosis stage was ≥3, the calculated amounts were 93.7%, 91.1%, 82.4%, and 95.9% for sensitivity, specificity, PPV and NPV, respectively. When fibrosis stage was more than four (≥4) sensitivity reached to 96.2%, specificity was 92.2%, PPV 55.5% and NPV 98.5% (table 4). Fibrosis stage of F≥1 has two degrees of freedom. Results of meta-analysis for sensitivity, specificity, positive predictive value, negative predictive value were summarized in table 5. In fibrosis stage of F≥2, degrees of freedom was 5. Maximum I-squared index was noted for sensitivity of 91.8%. Results of meta-analysis for fibrosis stage of F≥2 were indicated in table 5. The highest statistical heterogeneity was calculated 229.5 for PPV and the lowest amount was 14.8 for NPV. 

**Table 4:** Outcomes of meta-analysis; the results indicate the final accuracy of TE at different stages of fibrosis; for statistical analysis, the four stages of fibrosis according to Metavir and Brunt were considered equivalent. Confidence interval (CI), Positive Predictive Value (PPV), Negative Predictive Value (NPV), fibrosis (F)

**Biopsy stage**	**Sensitivity % (95% CI)**	**Specificity % (95% CI)**	**PPV% (95% CI)**	**NPV % (95% CI)**
F≥1	83.7% (78.6-88.7)	78.2% (72.8-83.6)	92.2% (88.7-95.7)	65.6 % (59.2-72.0)
F≥2	87.5% (85-90.1)	78.4% (75.2-81.7)	69.9% (66.3-73.5)	89.5% (87.1-91.9)
F≥3	93.7% (92-95.5)	91.1% (89-93.2)	82.4% (79.9-84.9)	95.9% (94.4-97.4)
F≥4	96.2 % (94.5-97.8)	92.2% (89.9-94.6)	55.5% (51.2-59.8)	98.5% (97.4-99.5)

**Table 5:** Results of meta-analysis for sensitivity, specificity, positive predictive value, negative predictive value on various level of fibrosis; Positive Predictive Value (PPV), Negative Predictive Value (NPV), fibrosis (F

	**Heterogeneity statistic**	**Degrees of freedom**	**P value**	**I-squared**	**Tau-squared**
F≥1, Sensitivity	0.22	2	0.897	0.0%	0.000
F≥1, Specificity	22.34	2	0.000	91.0%	307.52
F≥1,PPV	21.71	2	0.000	90.8%	191.84
F≥1,NPV	13.91	2	0.001	85.6%	186.66
F≥2, Sensitivity	60.85	5	0.000	91.8%	128.68
F≥2, Specificity	20.85	5	0.001	76.0%	55.402
F≥2,PPV	30.22	5	0.000	83.5%	114.18
F≥2,NPV	33.27	5	0.000	85.0%	55.201
F≥3, Sensitivity	79.12	6	0.000	92.4%	82.26
F≥3, Specificity	58.22	6	0.000	89.7%	71.80
F≥3,PPV	229.54	6	0.000	97.4%	514.27
F≥3,NPV	14.78	6	0.020	59.4%	6.78
F≥4, Sensitivity	30.33	3	0.000	90.1%	42.59
F≥4, Specificity	13.49	3	0.004	77.8%	19.07
F≥4,PPV	43.75	3	0.000	93.1%	302.15
F≥4,NPV	0.94	3	0.815	0.0%	0.000

The highest I-squared index was observed in PPV (F≥3). Degrees of freedom was six (F≥3) (table 5). Figure 2 (frost plot) (a,b,c,d) showed sensitivity (figure 2 a), specificity (figure 2b), PPV(figure 2 c) and NPV (figure 2d) test when F≥3. When F≥4, statistical heterogeneity for sensitivity was 30.33, specificity was 13.49, for PPV was 43.75 and for NPV became 0.94. Degrees of freedom were calculated 3 for all statistical indexes when F≥4. I-squared index was 93.1% and 90.1% for PPV and sensitivity, respectively (table 5). Figure 3 (frost plot) (a,b,c,d) revealed sensitivity (figure 3 a), specificity (figure 3 b), PPV (figure 3 c) and NPV (figure 3 d) when F≥4.

**Figure 2a:**
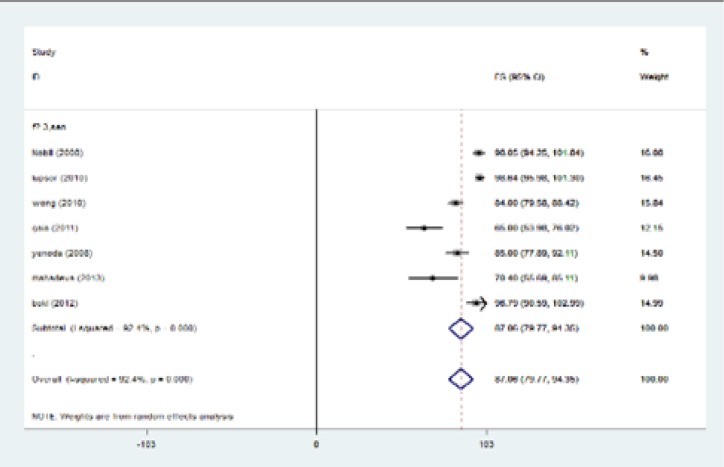
Sensitivity if F≥3, frost plot diagram

**Figure 2b:**
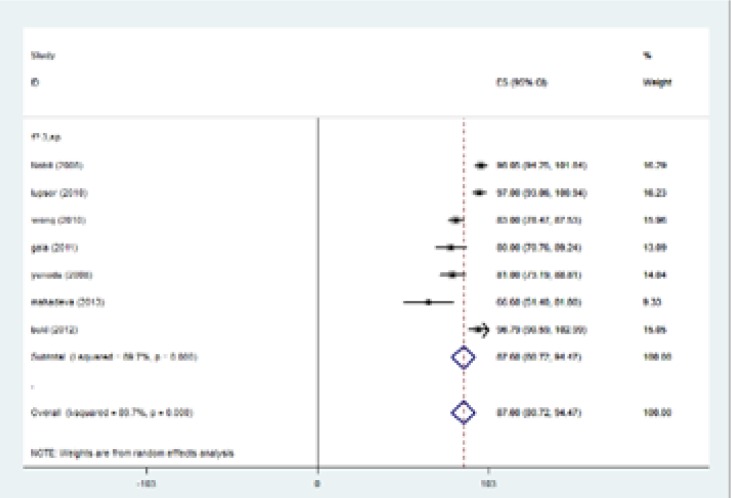
Specificity if F≥3, frost plot diagram

**Figure 2c:**
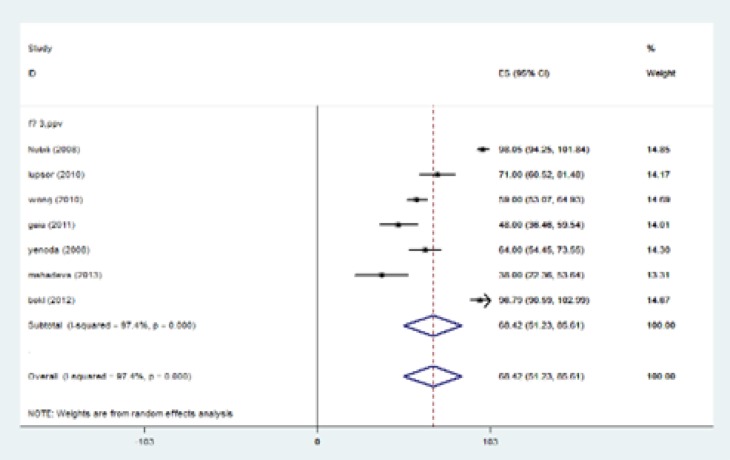
PPV if F≥3, frost plot diagram

**Figure 2d:**
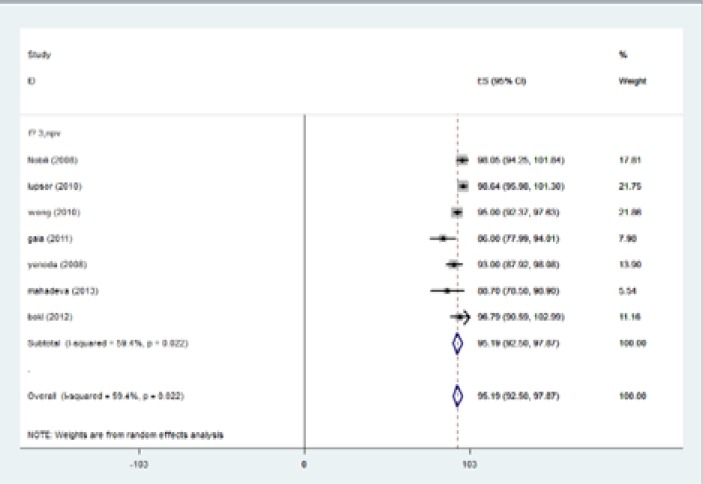
NPV if F≥3, frost plot diagram

**Figure 3a:**
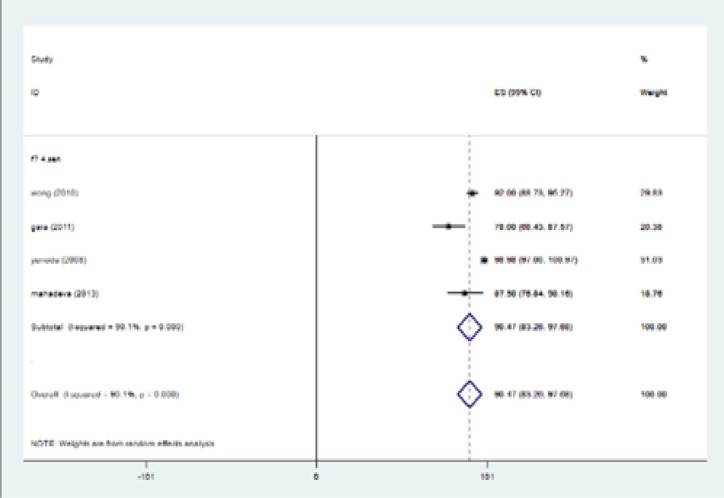
Sensitivity when F≥4, frost plot diagram

**Figure 3b:**
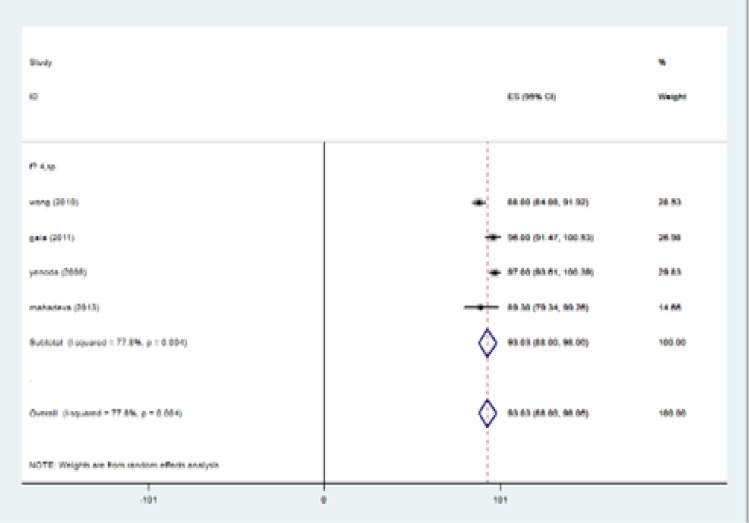
Specificity when F≥4, frost plot diagram

**Figure 3c:**
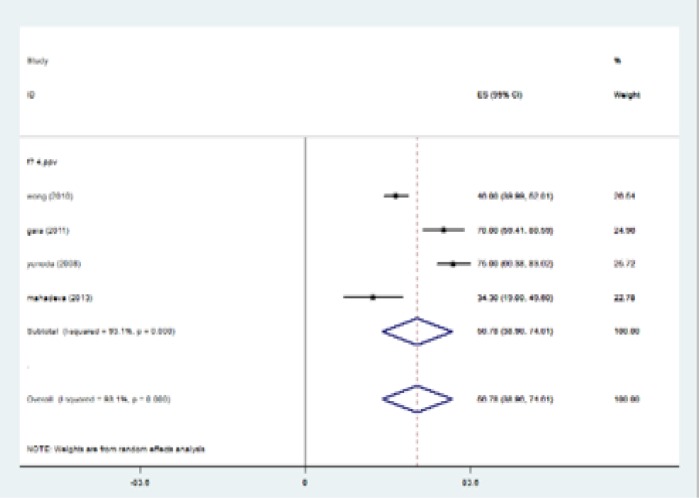
PPV when F≥4, frost plot diagram

**Figure 3d:**
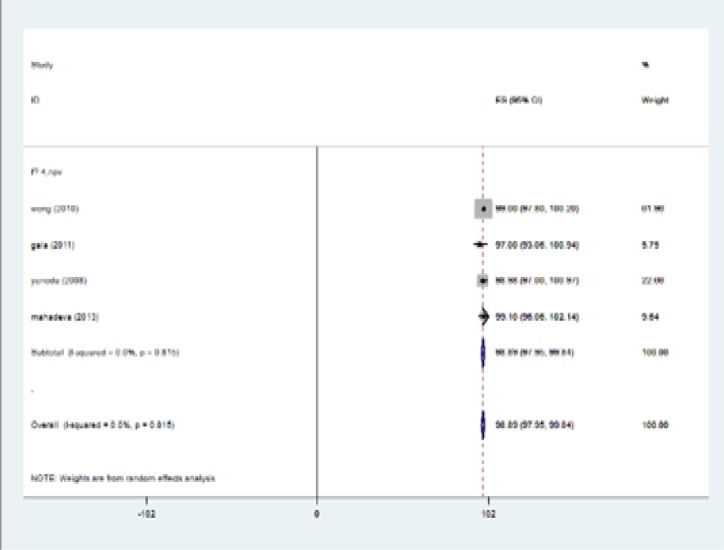
NPV when F≥4, frost plot diagram

## Discussion

In a meta-analysis study by Adebajo et al. (42). They examined the ultrasound-based TE for the diagnosis of hepatic fibrosis in recurrent hepatitis c virus after liver transplantation. Their finding showed that five papers examined fibrosis and indicated a sensitivity of 83% [95% confidence interval (CI): 77%-88%], and a specificity of 83% (95% CI: 77%-88%). Likewise with our results, in their practice the sensitivity and specificity of TE was high.

Chon et al. (43) performed a meta-analysis of 18 studies with 2772 chronic hepatitis B cases. They revealed that TE had high enough diagnostic accuracy for detecting liver fibrosis in patients with chronic hepatitis B. Similar to the present work, this article revealed the ability of TE in diagnosis of liver fibrosis although they did not evaluate the NAFLD patients. Chon et al. indicated that sensitivity and specificity for F≥2 were 74.3% and 78.3%, respectively. Predicting values for F≥3 were 74 and 63.8% and 84.6 and 81.5% for F≥4. Their results supported our findings from our research: as the fibrosis stage increased the accuracy of TE improved. In this regard, another meta- analysis reported by Talwalka et al. (44) showed that the sensitivity and specificity scores were 70 and 80.4 for F≥2, and 87 and 91 for F=4. Stebbing et al. (45) indicated the close amounts. In their study, chronic hepatitis C infection was the most common cause of fibrosis among the articles. 

They revealed that the pooled estimates for significant fibrosis (≥F2) had a sensitivity of 71.9% [95% CI: 71.4%-72.4%] and specificity of 82.4% (95% CI: 81.9-82.9%). Tsochatzis et al. (46) revealed the sensitivity and specificity scores of 79 (95% CI 0.74–0.82) and 78(95% CI 0.72–0.83) for F≥2, and 83 and 89 for F=4. The current investigation indicated that patients with fibrosis stage of ≥2 had the sensitivity of 87.5%, and specificity of 78.4%. While stage F≥4 had the sensitivity of 96.2% and specificity of 92.2%. Our data showed more accuracy of TE in the diagnosis of fibrosis, but this difference could be due to etiology of fibrosis, which means that we only investigated the NAFLD patients while the reason of fibrosis in other papers was different. 

Most of the mentioned papers discussed about the sensitivity and specificity of TE. In this particularity the present paper provided notable information about PPV and NPV of TE in detection of fibrosis. In a systematic review by J. K. Dowman et al. (47), they indicated that staging for NAFLD using a combination of radiology and laboratory procedures can decrease the requirement for invasive liver biopsy. Kwok R et al. revealed that the pooled sensitivities and specificities for TE to detect F ≥ 2, F ≥ 3 and F4 disease were 79% and 75%, 85% and 85%, 92% and 92% respectively. Like the current outcomes, they concluded that TE can exclude NAFLD patients with advanced fibrosis, primary evaluation is recommended to be done (48).

**Conclusion**

 TE has been validated in a wide spectrum of liver diseases like chronic hepatitis C (49-51), chronic hepatitis B (52-54), co-infection with human immunodeficiency virus (HIV) (55), alcoholic liver disease (56), primary biliary cirrhosis, and primary sclerosing cholangitis (57) and in the post-liver transplantation setting (58). These investigations indicated that TE was a valid method in the evaluation of fibrosis while liver biopsy was taken as the gold reference standard. Most of these papers revealed that TE was accurate enough to detect high stage of fibrosis and cirrhosis without consideration of baseline etiology (59, 60). Our results proved that as the fibrosis stage increases, the accuracy of TE improves. As a result, considering TE in advanced fibrosis will have more real results close to liver histology. Furthermore, TE can indicate the progress of liver fibrosis and would be an accurate procedure in the follow-up of these patients. 

The present study provided evidence that using TE in detecting level of fibrosis in NAFLD cases has high accuracy and can be a good alternative for liver biopsy in patients who cannot undergo invasive procedures. TE is an easy method to evaluate liver fibrosis, noninvasive, needing short time to obtain results appreciated by patients. Although further longitudinal investigations are needed to confirm the outcomes.

## Notes

**Authors’ Contribution**: Study concept and design: Seyed Moayed Alavian, Acquisition of data: Seyed Moayed Alavian, Seyyed Abbas Hashemi, Mohammad Gholami Fesharaki, Analysis and interpretation of data: Seyed Moayed Alavian, Seyyed Abbas Hashemi, Mohammad Gholami Fesharaki, Drafting of the manuscript: Seyed Moayed Alavian, Seyyed Abbas Hashemi, Critical revision of the manuscript for important intellectual content: Seyed Moayed Alavian, Statistical analysis: Mohammad Gholami Fesharaki, Administrative, technical, and material support: Seyed Moayed Alavian, Seyyed Abbas Hashemi , Study supervision: Seyed Moayed Alavian

## Notes

**Funding: **This study was supported by Baqiyatallah Research Center for Gastroenterology and Liver Diseases, Baqiyatallah University of Medical Sciences, Tehran, Iran.
